# Effect of Ethanol on Ag@Mesoporous Silica Formation by In Situ Modified Stöber Method

**DOI:** 10.3390/nano8060362

**Published:** 2018-05-24

**Authors:** Qian Chen, Yanling Ge, Henrika Granbohm, Simo-Pekka Hannula

**Affiliations:** Department of Chemistry and Materials Science, School of Chemical Engineering, Aalto University, 02150 Espoo, Finland; yanling.ge@aalto.fi (Y.G.); henrika.granbohm@aalto.fi (H.G.); simo-pekka.hannula@aalto.fi (S.-P.H.)

**Keywords:** core-shell, silver nanoparticles, silica mesoporous shell, ethanol effect

## Abstract

Tunable core-shell Ag@Mesoporous SiO_2_ spheres were synthesized via an in situ modified Stöber approach by varying the amount of ethanol (EtOH) expanding their potentials in many applications. Mesoporous silica was generated by adding tetraethyl orthosilicate (TEOS) to the mixture of colloidal Ag particles prepared by reducing silver nitrate (AgNO_3_) with L-ascorbic acid and using hexadecyltrimethylammonium bromide (CTAB) as a template at the presence of ethanol and sodium hydroxide (NaOH) at pH 10 as a catalyst. The average sizes of the Ag cores at the three increasing volumes of ethanol were ~47 ± 6, 36 ± 4, and 11 ± 5 nm, while the silica particle size and the thickness of the silica shells increased, resulting in a blueshift of localized surface plasmon resonances (LSPR) of the Ag NPs. The corresponding specific surface areas of silica particles were 356 ± 10, 419 ± 20 and 490 ± 25 m^2^ g^−1^, and average pore diameters varied from 5.7, 5.0 to 3.3 nm according to BET and BJH analyses. TEM studies confirmed the core-shell structure, pore sizes and shapes of mesoporous shells. The dissolution tests demonstrated that the release of Ag from the powder samples is pH-sensitive and time-dependent.

## 1. Introduction

Over the last several decades, silver nanoparticles (Ag NPs) have received considerable attention due to their unique properties [1,2,3,4]. For example, the optical property of Ag NPs is applied in localized surface plasmon resonance (LSPR)-based chemical and biological sensors, as well as LSPR-based substrates for surface-enhanced Raman scattering (SERS) and surface-enhanced fluorescence (SEF) [1,3,4]. Another increase in applications is use of Ag NPs as antibacterial agents [5]. The upsurge in bacterial resistance to antibiotics has promoted the need for extensive investigations on the antimicrobial properties of Ag NPs [5,6,7,8,9]. Despite the interesting properties of colloidal Ag NPs, the main drawbacks of their application are low dispersion stability, chemical and thermal reactivities [10,11], as well as decreased antimicrobial properties due to aggregation of Ag NPs [5,8]. Furthermore, some evidence of cytotoxicity of Ag NPs towards mammalian cells has also been reported [12]. To overcome the aforementioned limitations, various attempts have been made to design core-shell composites consisting of Ag NPs as a core in an inorganic shell. It was observed that the shells improve the colloidal stability of the Ag NPs cores, prevent their aggregation and concomitantly control the concentration of Ag ions required to achieve appropriate antimicrobial concentrations [1,10,11,12,13,14,15,16,17]. In particular, amorphous mesoporous silica shell (SiO_2_, pore size 2 to 50 nm) is an attractive candidate for controlled drug delivery system due to its large surface area, stable porous structure, high colloidal and chemical stability, as well as biocompatibility [10,11,12,17,18,19,20]. Owing to the LSPR of Ag NPs [1,4,21,22,23,24,25] and dielectric properties of silica [1,22,23,24,25], core-shell Ag@SiO_2_ nanocomposites could find use in emerging technologies, such as metal-enhanced fluorescence (MEF) for detection of chemical species in biochemistry and molecular biology [21,22,23], and shell-isolated nanoparticle-enhanced Raman spectroscopy (SHINERS) for investigation of various interfaces, especially in in situ conditions [24,25]. MEF uses fluorophore-Ag interaction to improve the fluorophores brightness and photostability [21,22,23], and SHINERS enhances Raman signal intensity also due to the LSPR of Ag NPs at any wavelength in the visible region of the spectrum [24,25]. Silica coating forms a controlled dielectric environment around Ag, raising precision in LSPR-based applications [1,22,23,24,25]. The performance of both technological designs can be varied by tuning the size and shape of the Ag cores and thickness of the SiO_2_ shells [21,23,24,25].

Typically, Ag@SiO_2_ composites are synthesized by Ag-seeded growth of silica through a Stöber method or a reverse microemulsion method [1,11,12,13,14,26]. The cores (Ag NPs) are stabilized with organic surfactants [1,10] or polymers [27,28]. Some coating processes involve use of coupling agents and/or surfactants, thus allowing deposition of silica onto the Ag cores [1,11,13,14]. The Stöber method involves hydrolysis and condensation of tetraethyl orthosilicate (TEOS) in the presence of alcohol and ammonia at room temperature, as shown in Equations (1)–(3) [29,30]. It has been shown that Stöber silica particles synthesized using ammonia as a catalyst forms uniform spheres, contrary to the acid-catalysed systems that result in gel structures [31]. However, this process typically results in dense structures with very small pore size and total pore volume, making them less favourable for potential applications in controlled drug delivery that demand porous carriers [32]. Many attempts have been made to achieve a desired surface morphology for silica by fine-tuning complex synthetic parameters in the Stöber method, such as temperature, ratio of reactants, nature of solvents and catalysts [14,29]. Although ammonia has typically been the most widely used catalyst, dimethylamine (DMA) has also been reported as a catalyst. DMA offers two advantages over ammonia: (i) DMA allows higher reproducibility for synthesizing silica shells due to its low vapour pressure and (ii) an appropriate amount of DMA prevents dissolution of silver and avoids formation of a [Ag(NH_3_)_2_]^+^ complex due to etching of the Ag core with ammonia [1,13,33]. However, Ag core etching is still observed in the presence of a high concentration of DMA [34].

Hydrolysis  Si(OR)_4_ + H_2_O ↔ HO–Si(OR)_3_ + ROH (1)

Alcohol condensation  (OR)_3_Si–OR + HO–Si(OR)_3_ ↔ (OR)_3_Si–O–Si(OR)_3_ + ROH (2)

Water condensation  (OR)_3_Si–OH + HO–Si(OR)_3_ ↔ (OR)_3_Si–O–Si(OR)_3_ + H_2_O (3)

R = alkyl group

The use of sodium hydroxide (NaOH) as a catalyst for silica layer formation has been shown to significantly prevent etching of Ag cores [1,20,34,35]. To date, very limited research has been executed on the preparation of Ag@mesoporous SiO_2_ composites using Ag NPs of size ranging from 20 to 100 nm with sodium hydroxide (NaOH) as a catalyst. Various alcohol cosolvents have been investigated using the Stöber method for producing silica of versatile size and surface morphology [36]. Ethanol as a low molar-mass alcohol cosolvent remains a key factor in changing the kinetics of the reactions for silica formation [37]. To the best of our knowledge, the effects of ethanol on the properties of both Ag core and silica shell of Ag@SiO_2_ particles has not yet been studied. Xu et al. [35] have synthesized core-shell Ag@SiO_2_ particles under acidic conditions using one-pot synthesis based on a modified Stöber method. However, their work presented no value for the surface area of the silica shell. We could not obtain porous silica using the same reaction conditions, despite the article claiming that it had produced a porous silica shell structure. In present work, we have made extensive modifications to Xu’s work, and found a correlation between the volumetric ratio of ethanol to water and the properties of Ag@SiO_2_ particles using NaOH as a catalyst. Additionally, the release of Ag from the synthesized samples was examined via pH-triggered dissolution tests.

## 2. Results and Discussion

### 2.1. Characterization of Core-Shell Ag@Mesoporous Silica Composites

**Figure 1:**
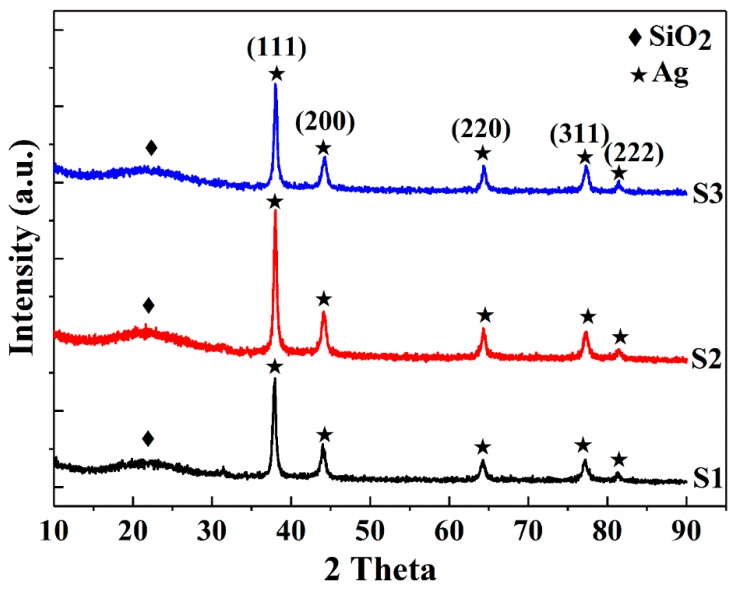
XRD patterns of S1, S2 and S3 samples.

To investigate the effect of ethanol on the Ag@SiO_2_ particle size and surface morphology, three samples S1, S2 and S3 were prepared in the presence of varying volumes of ethanol as a cosolvent (50 mL, 100 mL and 150 mL, respectively) using the Stöber method. Prior to investigating the ethanol impacts on the properties of the hybrid particles, XRD measurements were conducted to verify their structural and phase information. The XRD data (Figure 1) describe both amorphous and crystalline patterns. The broad humps in the diffraction spectra centered at 22° are due to amorphous silica particles, which are in good agreement with results presented in the literature [10,11]. Five sharp diffraction peaks related to the crystallized silver are indexed to be {111}, {200}, {220}, {311} and {222} reflections, corresponding to 2θ angles of 38.08°, 44.26°, 64.38°, 77.31° and 81.45°, respectively, a Fm-3m cubic structure of metallic silver (ICDD 04-014-0266). Silver was formed by ascorbic acid reduction of silver nitrate using CTAB as a cationic surfactant [35]. The average crystallite size (D) of Ag was calculated using Scherrer’s equation [38] (Equation (4)) and the strongest (111) diffraction peak:

(4) $$D=\frac{K\lambda }{\beta cos\theta }$$

where *K* denotes the shape factor = 0.89; λ = 1.5406 Å is the wavelength of the incident Cu *K*α radiation; β corresponds to the line broadening at full width at half maximum (FWHM) of the peak; and θ is the Bragg diffraction angle. The widths of the Bragg peaks represent the instrument-corrected broadenings to the diffraction peaks of Ag crystallites obtained by subtracting the instrumental broadenings. The crystallite sizes obtained for S1, S2 and S3 are 21, 24, 25 nm, respectively, showing that there are only minor differences in the Ag crystallite sizes of the samples.

**Figure 2:**
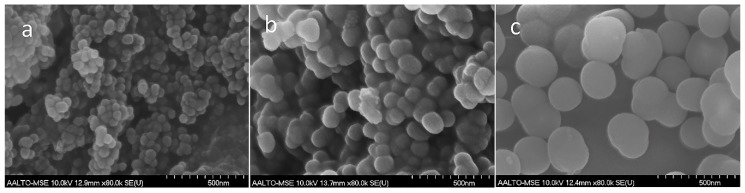
SEM images of surface morphology of dried powder samples: (**a**) S1, (**b**) S2, and (**c**) S3.

The volumetric impact of ethanol on the Ag@SiO_2_ particle size, surface areas and average pore sizes of the particles is tabulated in Table 1. The FE-SEM micrographs (Figure 2) demonstrate that the spherical silica particles have average diameters of 51, 105 and 219 nm for S1, S2 and S3, respectively. The standard deviations (STDEV) of the measured particle sizes for S1, S2 and S3 are 7, 15 and 28 nm, respectively, indicating a relatively broad variation in the particle size distributions. The size of silica spheres dramatically increases with increasing volume of ethanol. From Figure 2, it can also be seen that highly aggregated silica spheres were observed in both S1 and S2 (Figure 2a,b). Previous work [39] has shown that silica particle size in sol-gel synthesis is influenced by the relative concentration of water. The volumetric ratio of ethanol to water is 0.2, 0.4 and 0.6 for S1, S2 and S3, respectively. A higher concentration of water reactant promotes the rate of hydrolysis, but also decreases the rate of condensation reaction due to water formation (Equations (1) and (3)) [30]. This resulted in the production of smaller silica particles. Conversely, a lower concentration of water limits hydrolysis but enhances condensation. Thus, the primary particles produced through nucleation [40,41] will continue to grow either by oligomer addition [40] or solely through particles aggregation [41]. The presence of an ethanol solvent may promote esterification, which is the reverse direction of hydrolysis [42]. Increasing the concentration of ethanol decreases the rate of hydrolysis reaction. The sample obtained from the highest concentration of ethanol and lowest concentration of water has the greatest particle size due to a more hindered hydrolysis and enhanced condensation reaction.

**Figure 3:**
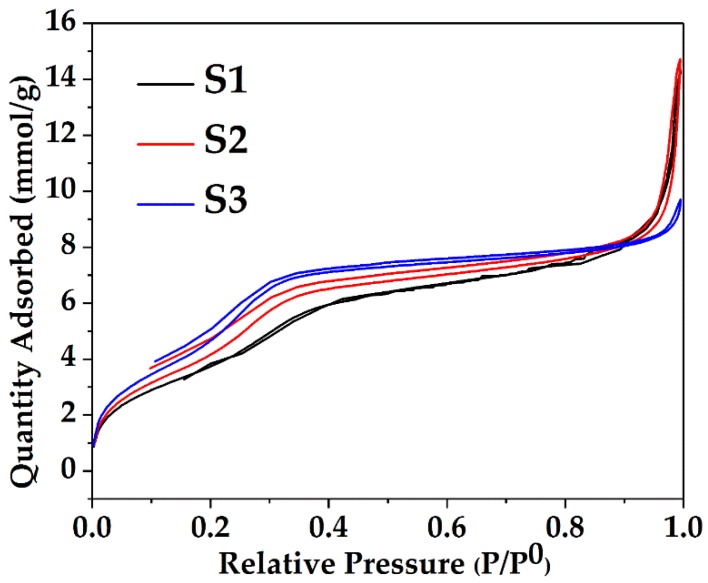
The adsorption/desorption isotherm of the synthesized Ag@SiO_2_ particles.

**Figure 4:**
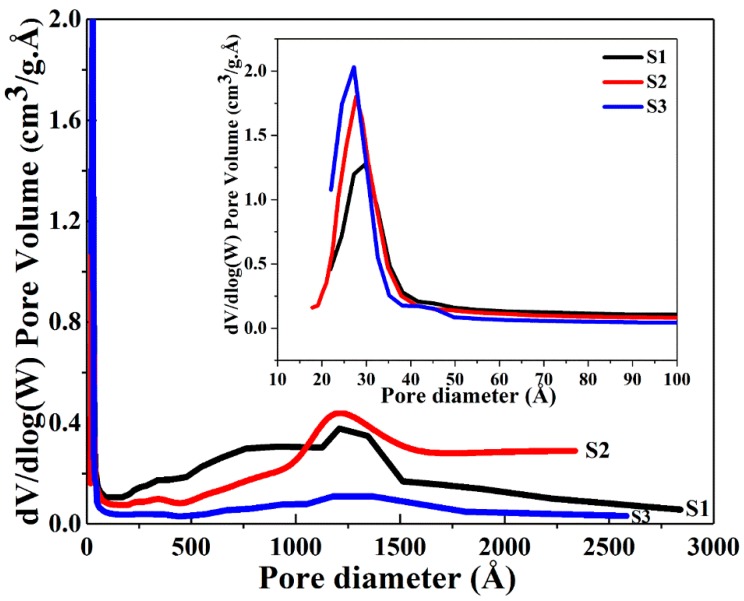
The pore size distribution of the Ag@SiO_2_ samples based on BJH desorption dV/dlog (W).

The BET data show that an increase in the surface area together with an overall concomitant decreasing trend in pore diameter and pore volume occurs when the volume of ethanol was increased from 50 to 100 and further to 150 mL. The concentration of 1 g CTAB at 250 mL water is 10.9 mM, which is much larger than its critical micelle concentration (CMC) in water (0.9 mM) [43,44], thus, micelles emerged. When the volume of ethanol was increased in the solvent mixture, the size of micelles gradually decreased due to the stronger interaction between the CTAB tail group and ethanol [43]. Increasing volumes of ethanol led to the smaller pore size found in the growing silica network encasing the CTAB micelles. Figure 3 and Figure 4 present the nitrogen adsorption-desorption isotherms and pore size distributions of the dry powders. All samples exhibit an approximate type IV isotherm pattern (Figure 3), as defined by IUPAC [45,46,47] for mesopore characteristics. Type H3 hysteresis loops were observed in S1 and S2, indicating slit-shaped pores (Figure 3) and a hysteresis loop of type H4 displayed in S3 associated with narrow slit pores [46] (Figure 3). A high uptake of the adsorption isotherms at the relative pressure (P/P^0^) above 0.9 was observed for S1 and S2, and the H3 type isotherm patterns of S1 and S2 are slightly broader than the H4 type of S3. This observation suggests that with less volume of ethanol, the capillary condensation shifts to a higher relative pressure, thereby inducing larger pores. This trend is consistent with the average pore diameters of 5.7, 5.0 and 3.3 nm for S1, S2 and S3 evaluated by BJH desorption data. Figure 4 shows the broad distribution of pores observed predominantly below 6 nm in all samples. The narrow, high uptakes, ranging between 2–5 nm can be attributed to the internal pores of primary silica particles, while the long weak tails are associated with the voids between the interparticles or aggregations of the particles.

**Figure 5:**
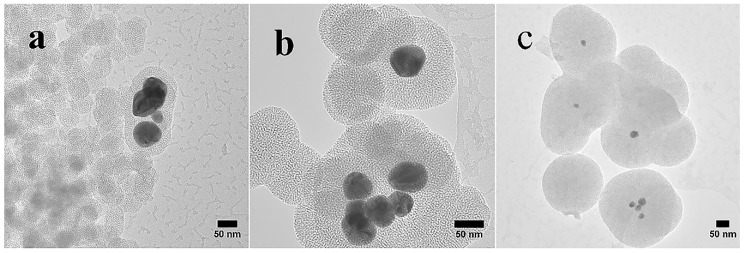
TEM images showing the surface morphology of both core-shell Ag@SiO_2_ and core-free silica particles: (**a**) S1 with 50 mL ethanol, (**b**) S2 with 100 mL ethanol and (**c**) S3 with 150 mL ethanol.

The TEM images (Figure 5) reveal the round mesoporous core-shell structures of synthesized particles along with core-free particles. Both shells and core-free particles display a porous character, in other words the pore sizes and shapes are not affected by the Ag cores. Silver particles appear darker in the core, as the electron density of Ag is much higher than that of SiO_2_ [13]. As described earlier, the Ag NPs were formed by reduction of silver nitrate using ascorbic acid as the reducing agent and CTAB as the cationic surfactant [35]. During generation of SiO_2_ on Ag NPs, core-free SiO_2 _particles that are devoid of Ag cores were produced simultaneously, and some non-coated silver particles were also present. The CTAB was removed by washing the wet samples with ethanol and drying. The average sizes of the Ag particles estimated from the Ag@mesoporous SiO_2_ particles based on the TEM images with standard deviations are found to be ~47 ± 6 nm, 36 ± 4 nm and 11 ± 5 nm, while the calculated average thickness of the silica shells obtained from the measured core-shell particles are ~2 nm, 35 nm and 104 nm for S1, S2 and S3, respectively. The finding that each core-shell particle consisted of one or more Ag crystallites is in accord with the XRD measurements. During the formation of Ag NPs in the synthesis process, the bare Ag nanocrystals without the silica shell may grow into larger Ag crystallites in the aggregates. Therefore, the size of Ag crystallites in the aggregates may be larger than the size of the Ag crystallites cores embedded in silica, as observed in XRD evaluations for S3. A decreasing trend in the size of Ag cores along with the growing thickness of the silica shells found for increasing volumes of ethanol is depicted in Figure 5. Bae et al. [26] found that the size of Ag cores is dependent on the micelle size, nature of solvent and the concentration of the reagents. The as-synthesized Ag nuclei diffuses through the CTAB micelles. As was mentioned earlier, the size of micelles decreases with increasing volume of ethanol, thus resulting in smaller Ag particles.

Single or multiple Ag cores are entrapped in SiO_2_ shells, as well as core-free silica particles are present in all samples. Furthermore, for core-shell particles, single small Ag cores appear to be more dominant in S3 than in S1 and S2. The images (Figure 5) confirm that the Ag cores were not etched away, a finding contrary to some earlier observations for ammonia catalysed synthesis, where hollow spheres were observed around Ag particles [34]. CTAB functions as both a stabilizer and a template [20,48], as well as prevents the newly formed Ag from rapid aggregation [10]. CTAB assembles with SiO_2_ via two routes: (i) the positive polar head group of CTAB neutralizes the negatively charged silica (due to the presence of silanol group Si-OH on the particle surface); and (ii) the interaction between the hydrophobic tails of CTAB makes the aggregation of SiO_2_, hence, the silica grows in size [49]. Han et al. [20] proposed a growth model of Ag cores in Ag@MSN, due to the low degree of silicate polymerization, the Ag NPs distributed all over the silica shell could transfer and in turn aggregate into larger single or multiple Ag cores. Then the subsequent removal of CTAB with ethanol and heating led to the formation of Ag cores encapsulated in the mesoporous silica shells. The relatively broad variation in the size of silica between the TEM and SEM studies can be attributed to the limited number of particles in the TEM samples.

**Figure 6:**
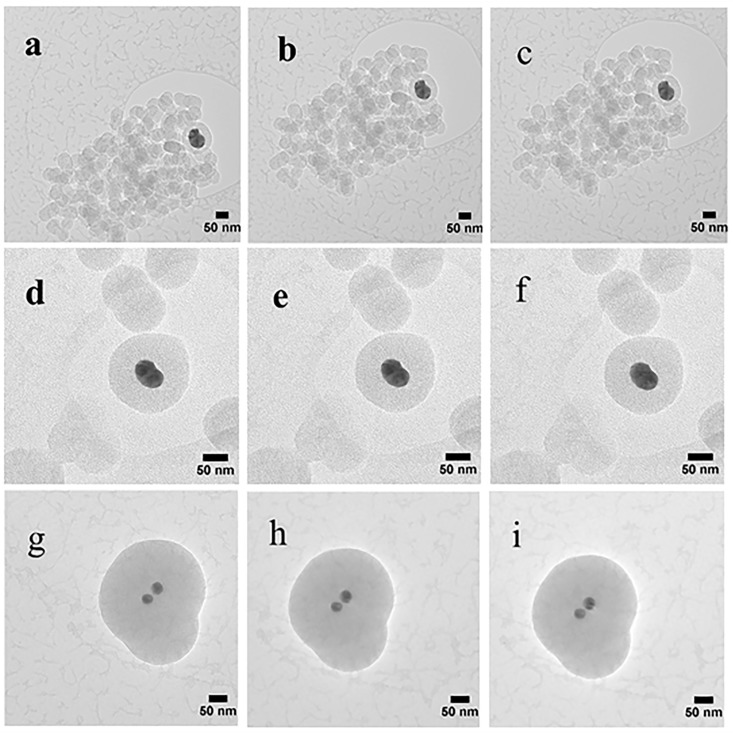
TEM images showing the morphology of the core-shell Ag@SiO_2_: S1 images taken at (**a**) 0 s, (**b**) 75 s, and (**c**)130 s; S2 images taken at (**d**) 0 s, (**e**) 16 s, and (**f**) 92 s; S3 images taken at (**g**) 0 s, (**h**) 86 s, and (**i**)162 s.

**Figure 7:**
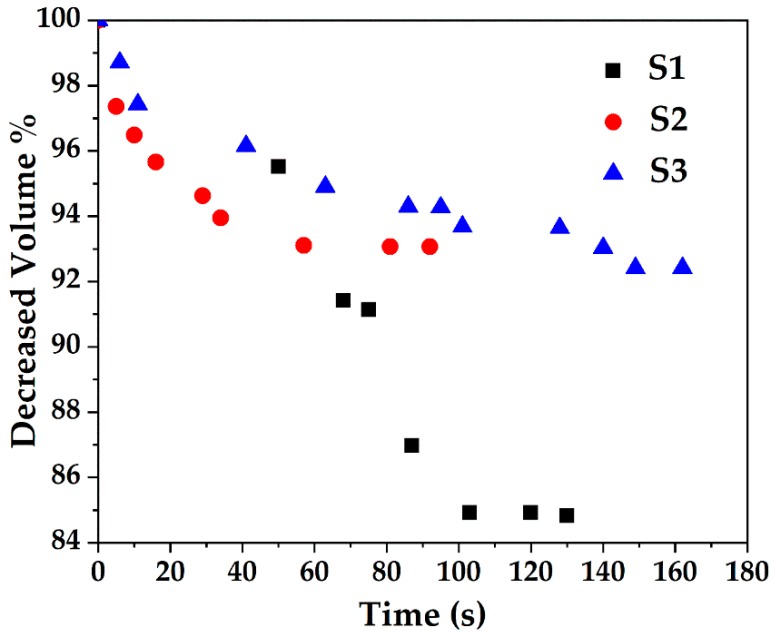
Decrease in volume of Ag@SiO_2_ samples of S1, S2 and S3 due to exposure to electron beam as a function of time.

The samples were also subjected to a series of TEM studies at various time intervals to study their structural changes under the electron beam. TEM imaging was performed at ambient temperature under 200 kV, and images were taken with a short exposure time of 0.1 s. It was observed that the porous silica shells shrank into denser structures under the high energy emitted by the electron beam. Initially, all samples with slit-shaped pores appeared distinctly. As time passed, densification occurred as denser structures were observed due to shrinkage of the pores (Figure 6). The change in volume as a function of time is depicted in Figure 7. The core-shell particles are assumed to be symmetrical spheres for the volume evaluations. As can be seen, S1, S2 and S3 underwent a decrease in volume of 15% (0–130 s), 7% (0–92 s) and 8% (0–162 s), respectively. Based on the initial concentration of AgNO_3_, TEOS and measured total pore volume, the pore volume fractions of S1, S2 and S3 were found to be 54.3%, 55.2% and 46.2%, respectively. It is worth noting that 0 s was counted after the images were well focused. The electron beam elevated the temperature on the silica surface, thus inducing densification of silica layers during shrinkage of the pores [50]. Furthermore, the pore sizes of S1, S2 and S3 (Figure 6) were estimated by TEM FFT, showing a decreasing trend in the average values of 4.75 nm, 4.35 nm and 3.95 nm, respectively. This behaviour further confirms the porous structure of the obtained particles and is consistent with the BET results.

**Figure 8:**
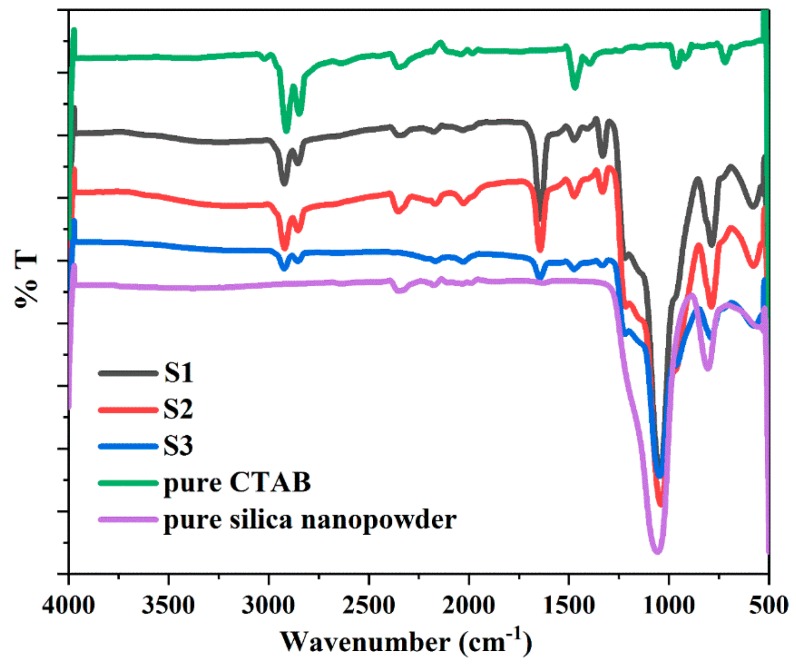
FTIR spectra of pure CTAB and synthesized samples S1, S2 and S3.

The FTIR spectra of the synthesized samples show the characteristic bonds corresponding to the structure of core-shell materials, in comparison to CTAB and pure silica nanopowders (Figure 8). All samples exhibit nearly identical characteristic bonds. The absorption bands around 2920 and 2849 cm^−1^ appear in all synthesized samples, corresponding to the CH_2_– stretching vibrations of CTAB (asymmetric at 2920 cm^−1^ and symmetric at 2849 cm^−1^) [51,52]. This indicates that CTAB residues remained inside some of the internal pores of the silica and were not completely removed after washing and drying. A decrease from S1 to S3 spectra associated with CTAB (2920 cm^−1^ and 2849 cm^−1^) signifies a decreasing amount of CTAB residues due to the employment of increasing volumes of ethanol in the syntheses. The spectra appearing around 2354 cm^−^^1^ in S1, S2, CTAB and pure silica are attributed to the artefacts from the device. Water shows an intense characteristic band at around 1646 cm^−1^, which is assigned to the bending vibration of the hydroxyl group of the adsorbed water on silica [53,54,55]. The strong decrease in the intensity of the spectra (1646 cm^−1^) from S1 to S3 is a result of the decreasing amount of adsorbed water. The 1475 cm^−1^ band originated from the C–H bending vibration of CTAB residues [56]. The intense and broad bands at 1045 and 790 cm^−1^ correspond to the asymmetric and symmetric stretching vibrations of Si–O–Si bonds, which identify the presence of SiO_2_ [37,53,57]. The 969 cm^−^^1^ band can be assigned to Si–O in-plane stretching vibrations of the silanol (Si–OH) groups [53,55]. The hydrophilic feature of the Si–OH groups permits the surface adsorption of water, thus confirming the spectrum of the hydroxyl group at 1646 cm^−1^. The 580 cm^−1^ band presents the Si–O stretching of the SiO_2_ network defects [30,55].

**Figure 9:**
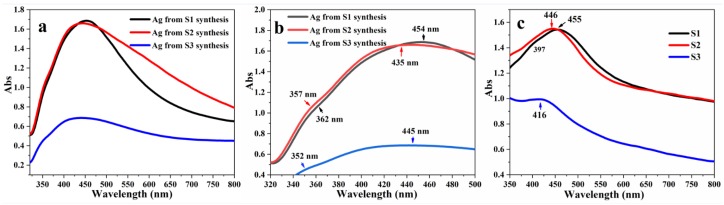
UV-vis spectrum of (**a**) colloidal Ag NPs from synthesis process of S1, S2 and S3 and (**b**) enlargement of Figure 9a and (**c**) dried samples of S1, S2 and S3.

The colloidal Ag NPs produced following the synthesis process of S1, S2 and S3 without the addition of TEOS were subjected to UV–Vis measurements (Figure 9a,b). The UV–Vis absorption spectra of dried samples S1, S2 and S3 with a loading of 0.5 mg/mL Ag@SiO_2_ in water are shown in Figure 9c. As shown in Figure 9a,b, the LSPR peaks of the bare colloidal Ag NPs are located at 362 nm, 357 nm and 352 nm for the S1, S2 and S3 synthesis processes, respectively. This observation demonstrates that the position of the Ag NPs absorption peak depends on the size of the Ag particles. In this study, a decrease in the Ag size of both bare Ag and Ag cores from S1 to S3 was observed, at the same surrounding medium, a blueshift in the peak positions arose from a change in particle permittivity [58]. Ag with a plasmon absorption peak in the range of 300–450 nm has been shown to be the most common choice for coupling with the fluorophores [22]. The CTAB capped Ag NPs redshifted to 454 nm (S1), 435 nm (S2) and 445 nm (S3) with respects to the peaks for the uncoated Ag NPs. After generating different thicknesses of silica onto the Ag cores, the observed LSPR absorption bands for dried samples S1, S2 and S3 were observed to have broadened as well as shifted to 455 nm, 446 nm and 416 nm, individually. Compared with the values of LSRP from the bare Ag NPs, the redshifts appear to be caused by condensation of SiO_2_ onto the Ag NPs, leading to an increase in the local refractive index around the Ag NPs, indicating the formation of core-shell Ag@SiO_2_ particles [1,14]. As observed in the TEM images, an increase in the thickness of the SiO_2_ shell occurred with increasing volume of ethanol. As the thickness of SiO_2_ shell grew, scattering tended to dominate and induce blueshifts between S1, S2 and S3 (S1 > S2 > S3). It was also noted during the TEM observation that aggregated bare Ag NPs was present in S1, having absorption peak at 397 nm. The position of absorption shifted from 362 to 397, probably due to aggregation of Ag NPs after drying.

### 2.2. Detection of Ag Release from the Powder Samples

**Figure 10:**
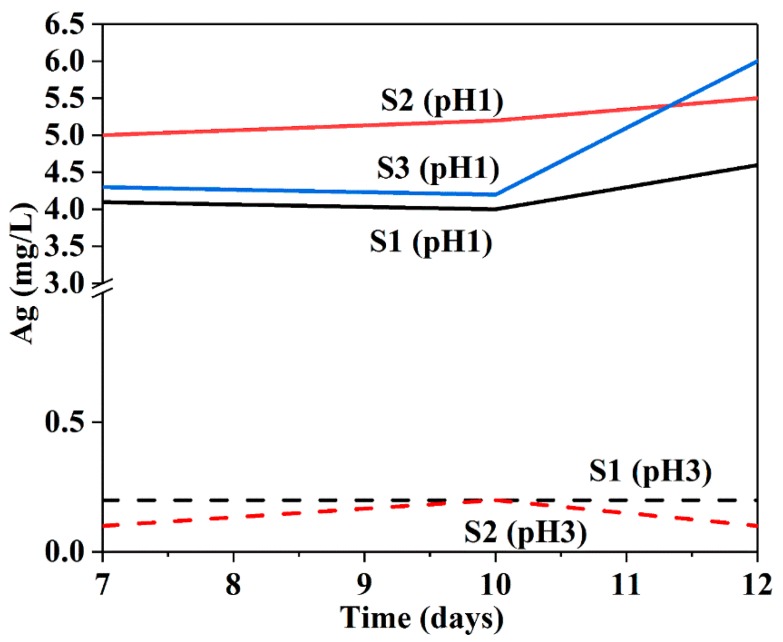
Release of Ag from S1, S2 and S3 at 7, 10 and 12 days under pH 1 acidic condition.

The ICP-AES was used to determine the concentration of released Ag-species (Ag NPs and Ag^+^) from the powder samples at different pH values. As shown in Figure 10, at pH 1, the release of Ag ions for all samples remained almost constant for 7 to 10 days, followed by an increase in release at 10–12 days. This may be ascribed to partial etching of internal pores in silica shells, leading to enhanced diffusion of Ag ions, as the silica slowly dissolved at pH 1 in a HNO_3 _solution for longer periods [59]. Although the initial release values for the Ag species are not comparable due to the presence of uncoated bare Ag NPs in all samples, the increases in fast release rates over 10 days were observed for all samples, particularly for S3 due to its larger surface area, indicating the release of Ag from the cores. At pH 3, a release of 0.1–0.2 mg L^−1^ Ag for sample S1 was measured for all times, while the release of S2 and S3 remained below the detection limit (0.1 mg L^−1^). At pH 5, all the samples had releases below the detection limit. There is a big difference between the amount of dissolved Ag ions at pH 1 and 3. These results clearly indicate a pH-sensitive release behaviour in the samples: pH 1 appears to be the most effective trigger, whereas pH 5 restricts the release of Ag ions. Furthermore, the release behaviour depends on the amount of time, as demonstrated by the longer period of acid administration.

## 3. Materials and Methods

### 3.1. Materials

For the preparation of silver nanoparticles, silver nitrate (AgNO_3_, ≥99.0%, Sigma-Aldrich, St. Louis, MO, USA), L-Ascorbic acid (C_6_H_8_O_6_, 99.0%, Sigma-Aldrich, St. Louis, MO, USA), and hexadecyltrimethylammonium bromide (C_19_H_42_BrN, CTAB, ≥99.0%, Sigma-Aldrich, St. Louis, MO, USA) were used as the silver precursor, reducing agent, and template for the preparation of silver nanoparticles, respectively. Tetraethyl orthosilicate (Si(OC_2_H_5_)_4_, TEOS, ≥99.0%, Sigma-Aldrich Chemie GmbH, Steinheim, Germany), de-ionized water (H_2_O), and ethanol (C_2_H_6_O, ≥99.5%, Altia Oyj, Helsinki, Finland) were used as the silica precursor, reagent, and cosolvent, respectively. Sodium hydroxide (NaOH, 0.1 M, Sigma-Aldrich Chemie GmbH, Steinheim, Germany) was used as the catalyst for the formation of silica. Pure silica nanopowder (SiO_2_, >92.7% PlasmaChem GmbH, Berlin, Germany) was used as a reference sample in the FTIR measurements.

### 3.2. Preparation of Core–Shell Ag@SiO_2_ Particles

Core-shell Ag@SiO_2_ were obtained using a modified Stöber method [35] by varying the volume of ethanol in the presence of a higher concentration of CTAB as well as a prolonged mixing time. Unlike the acidic condition proposed by Xu et al. [35], an alkaline condition was carried out in this work, along with the higher concentration of CTAB and an appropriate mix of ethanol, to achieve various porous structures of silica. Initially, Ag NPs were obtained by reducing AgNO_3_ with L-ascorbic acid. 10 mL of freshly prepared 0.1 M AgNO_3 _was added into 250 mL water containing 1 g CTAB under vigorous mixing. Next, 20 mL of 0.5 M L-Ascorbic acid was titrated into the solution. The mesoporous silica shell was then generated onto the formed Ag NPs as follows: three different volumes of ethanol, namely 50, 100 and 150 mL, were added to three white colloidal solutions containing CTAB and Ag NPs, respectively. Thereafter, the pH was adjusted to 10 by adding 0.1 M NaOH to each colloidal solution, which their colour changed from white to green-brown, indicating the formation of Ag NPs. This was followed by the dropwise addition of 2.5 mL of TEOS to each mixture. NaOH was further added to keep the alkaline reaction conditions constant, as the pH of the reaction mixtures decreased with the addition of TEOS. The mixtures were then left to react for 6 h and further aged under ambient conditions for 12 h. The products were washed three times with ethanol, collected by centrifugation, and then dried at 100 °C for 1 h followed by further drying at 150 °C for 3 h. Six syntheses were carried out for each set of samples. After drying, the samples were mixed in order to collect sufficient dry powder from each sample. The obtained dry samples were marked as S1, S2 and S3, respectively.

### 3.3. Characterization

The surface morphology and size of the synthesized particles were investigated with secondary electron imaging on a Cold Field Emission Gun Scanning Electron Microscope (Hitachi S-4700 FEG-SEM, Tokyo, Japan) that was operated at an accelerating voltage of 10 kV. The mean particle size was evaluated from the SEM images using ImageJ software (Image J 1.45b) from a minimum of 300 particles. The Ag@SiO_2_ structure was detected using Transmission Electron Microscopy (TEM, Tecnai G2 F20 FEG, FEI Hillsboro, OR, USA) with an accelerating voltage of 200 kV. Analysis of the TEM images was performed using the Gatan DigitalMicrograph^TM^ software (Gatan Inc., Pleasanton, CA, USA), and the pore size was estimated by FFT (fast Fourier transform). The core-shell Ag@SiO_2_ structures were verified by UV–vis absorption spectroscopy in the range of 300–800 nm with a Hitachi U-5100 UV/VIS spectrophotometer (Tokyo, Japan). The characteristic functional groups of core-shell particles were identified by Fourier Transform Infrared Spectroscopy (FTIR) at the spectral range of 500–4000 cm^−1^ on a Nicolet 380 FTIR spectrometer (Thermo Fisher Scientific, Waltham, MA, USA). The specific surface areas (SSA) were measured with nitrogen adsorption-desorption isotherm via the Brunauer-Emmett-Teller (BET) method. Prior to the measurements, the samples were degassed at 180 °C for 3 h. The average pore sizes, total pore volumes and pore size distributions were obtained with nitrogen desorption data using the Barrett-Joyner-Halenda (BJH) model. Both were carried out with a Micromeritics Tristar II 3020 (Micromeritics Instrument Corp., Norcross, GA, USA) using nitrogen gas as an adsorbate at 77 K. The phase composition and crystal structure of the synthesized particles were determined by X-ray Diffraction (XRD, PANalytical X’Pert Powder Pro, Malvern Panalytical, Almelo, The Netherlands) using Cu Kα_1/2_ radiation (λ_α1_ = 1.5406 Å) in the range of 10–90° (2θ). A standard silicon was measured to determine the instrumental broadening in order to calculate the average crystallite sizes of silver based on the XRD data using Scherrer’s equation [38]. Analysis of the XRD patterns was performed using X’Pert HighScore Plus Software (Malvern Panalytical, Almelo, The Netherlands). The silver concentrations from the dissolution tests were determined by Inductively Coupled Plasma Atomic Emission Spectroscopy (ICP-AES) using a Perkin Elmer ICP-AES Optima 7100 DV (Perkin Elmer, Waltham, MA, USA). The measurement error was estimated as ±5%. The silver emission spectra were measured at 328.068 nm and 338.289 nm.

### 3.4. Release of Silver from the Powder Samples

The pH-triggered dissolution tests were performed to detect the release of Ag species. The sample powders were dispersed by ultrasonication for 1 h at a concentration of 4 mg/mL, i.e., a total loading of 100 mg in 25 mL of pH 1, pH 3 and pH 5 HNO_3_ solution, respectively. The samples were prepared in triplicates, with the exception of S3 samples at pH 3 for 7, 10, and 12 days, and S3 sample at pH 5 for 7 days, which were performed in duplicates. Ag release behaviour was investigated for 7, 10 and 12 days. The dispersions were filtered through 0.45 μm Nylon syringe filters to remove solid particles. Then, 10 mL of the filtered solution was taken and acidified with 1 mL of concentrated HNO_3_, after which the Ag concentration was analysed using ICP-AES. The reported values represent averages from the measurements under each condition.

## 4. Conclusions

A tunable size and morphology for Ag@Mesoporous SiO_2_ was achieved via a one-pot synthesis based on a modified Stöber process by varying the volume of ethanol. The use of sodium hydroxide as a catalyst prevents the etching of Ag cores. The nuclei aggregations and the CTAB-stabilized surface interactions allow single or multiple Ag seeded SiO_2_ growth as well as the generation of core-free silica particles. Ethanol as a cosolvent plays a key role in adjusting the size of the CTAB micelles, thus influencing the size of the Ag cores. The ratio of ethanol to water impacts the kinetics of the hydrolysis and condensation reactions for mesoporous silica shells generation. Our studies offer control of the specific surface area, pore size and thickness of the SiO_2_ coating independently of the size of the Ag cores, thus broadening their potential in versatile applications. For example, Ag@SiO_2_ particles providing Ag cores of not only larger size (>20 nm) but also different thicknesses and porosity of the silica shells can offer interesting materials as nanoresonators in SHINERS studies, as well as probes for MEF applications when coated with a thin layer of fluorescent dye. Due to the variability of the structural characteristics in the studied samples, i.e., the co-existence of uncoated bare Ag NPs, core-free silica and core-shell Ag@Mesoporous SiO_2_ particles, the delivery of silver between samples requiring up to 10 days may be difficult to compare. Nevertheless, all the samples demonstrate pH-sensitive and time- dependent release behaviour, which are crucial for antibacterial applications.

## Figures and Tables

**Table 1:** The properties of dried samples prepared with different volumes of ethanol via a modified Stöber method (STDEV = standard deviation).

Samples	Ethanol (mL)	Particle Size ± STDEV (nm)	Specific Surface Area ± STDEV (m^2^ g^−1^)	Average Pore Diameter (nm)	Pore Volume (cm^3^ g^−1^)
S1	50	51 ± 7	356 ± 10	5.7	0.54
S2	100	105 ± 15	419 ± 20	5.0	0.56
S3	150	219 ± 28	490 ± 25	3.3	0.39
